# Editorial to the Special Issue on “Visible Light Communications, Networking, and Sensing”

**DOI:** 10.3390/s21124004

**Published:** 2021-06-10

**Authors:** Stanislav Zvánovec, Zabih Ghassemlooy, Rafael Perez-Jimenez, Luis Nero Alves

**Affiliations:** 1 Department of Electromagnetic Field, Faculty of Electrical Engineering, Czech Technical University in Prague, 16627 Prague, Czech Republic; 2 Optical Communications Research Group, Faculty of Engineering and Environment, Northumbria University, Newcastle upon Tyne NE1 8ST, UK; z.ghassemlooy@northumbria.ac.uk; 3 Institute for Technological Development and Innovation in Communications (IDeTIC), Universidad de Las Palmas de Gran Canaria (ULPGC), 35017 Las Palmas de Gran Canaria, Spain; rafael.perez@ulpgc.es; 4 Instituto de Telecomunicações and Departamento de Electrónica, Telecomunicações e Informática, Universidade de Aveiro, 3810-193 Aveiro, Portugal; nero@ua.pt

The next generation wireless technology networks and beyond (i.e., fifth and sixth generation) are changing the global landscape for the interconnectivity of everything-to-everything. To this end, different communication technologies, including millimeter wave, terahertz, and optical wireless, are to be utilized. The latter includes the three spectral bands of ultraviolet, visible, and infrared. Recently, we have seen a growing interest in the potential use of the visible band in the front end of the access networks, mostly in indoor environments. The system is better known as visible light communications (VLC)—also referred to as LiFi, which offers new opportunities by simply using the white light-emitting-diode (LED)-based lighting installations as the transmitter (i.e., an optical antenna). LEDs have a longer life expectancy, a higher tolerance to humidity, much higher switching speeds (orders of magnitude higher), lower costs, and lower power consumption, as compared to traditional incandescent and fluorescent lights, which are being phased out gradually. Recent advances in solid-state technologies have made highly energy-efficient and high-speed LEDs available for illumination, data communication, indoor localization, and sensing in a range of applications, in both indoor and outdoor environments. To give an insight into the latest research, innovations, and advances being made, as well as the technologies, applications, challenges, and solutions proposed, this Special Issue brings together 22 original research and review articles in the field of VLC systems. These manuscripts were selected based on the following topic lines—new techniques and modulations; VLC channel characterization, optical camera-based communication, visible light positioning, and underwater and vehicular applications. We hope that these papers will be of interest to those working in this interesting, new, and challenging area as part of the emerging 4th Industrial Revolution. The papers are grouped into five parts, as follows.

The first part is devoted to the development of new VLC techniques. J. Bruycker et al. [1], in their paper “*Polarization Differential Visible Light Communication: Theory and Experimental Evaluation*”, proposed and experimentally demonstrated a differential polarization modulation scheme, using orthogonal polarizers at both the transmitter and receiver ends of the VLC link. A general method to evaluate the SNR as a function of the receiver rotation with regard to the transmitters is determined and experimentally confirmed by measurements.

In the next paper, “*A 40 Mb/s VLC System Reusing an Existing Large LED Panel in an Indoor Office Environment*”, X. Li et al. [2] provide details of a novel experimental system based on an off-the-shelf LED panel typically used in office environments, with dimensions of 60 × 60 cm^2^. The low-complexity multi-carrier version of carrier-less amplitude and phase modulation (mCAP) is used to exploit the spectrum out of the 3-dB bandwidth of the LED, and a DSP-based solution is proposed to remove the baseline wander effect, due to flickering in the VLC system.

Following two papers dealing with LED characterization, M. Galal et al. [3], in his paper on “*Experimental Characterization of RGB LED Transceiver in Low-Complexity LED-to-LED Link*”, proposes a low-complexity and energy-efficient LED-to-LED communication system, using off-the-shelf red-green-blue (RGB) LEDs, where the red sub-LED is employed as a photodetector in the photovoltaic mode, while the green sub-LED is the transmitter. Authors derive a formula for the LED photodetector response at varying distances, and propose a matched filter technique to increase the error-free distance of the system. J. Sticklus et al. [4], in “*Experimental Characterization of Single-Color Power LEDs Used as Photodetectors*”, analyze the spectral, temporal, and spatial characteristics of two single-color power LED series employed as the PD, as compared to a common Si PIN photodetector. The dual use of the same LED as an emitter and detector is discussed with respect to wavelength dependence and practical issues, with further filtering proposed.

The next papers are devoted to modulation formats. L. Rodrigues et al. [5], in the paper on “Optimized Analog Multi-Band Carrierless Amplitude and Phase Modulation for Visible Light Communication-Based Internet of Things Systems”, develop a VLC-based IoT system architecture based on mCAP modulation, with the emitter implemented in the digital domain, while the demodulation in the receiver is accomplished in the analog domain. The authors focus especially on the filtering aspects, and the usage of guard bands parameters is evaluated. In the paper on “Advanced Modulation Format of Probabilistic Shaping Bit Loading for 450-nm GaN Laser Diode based Visible Light Communication”, G. Li et al. [6] focus on probabilistic shaping bit loading discrete multi-tone modulation, to improve the spectral efficiency and system performance. This is experimentally validated, through realization of a 10.23 Gbps laser diode-based transmitter, over a 1.2-m long transmission span, with a signal bandwidth of 1.5 GHz.

The next three papers provide more insight into the simulations of specific channel parameters and unique systems. Z. Nazari Chaleshtori at al. [7], in the paper “*Utilization of an OLED-Based VLC System in Office, Corridor, and Semi-Open Corridor Environments*”, focus on the unique organic LED panel transmitters placed on walls in various indoor environments, based on the 3D ray tracing simulations that the new statistics of root-mean-square delay the spread, from which optical path losses are derived. The next paper by S. Stainton et al. [8] on “*A Loss Function for Training Neural Networks in Communication Systems*” proposes a novel error vector magnitude (EVM) loss method, which seeks to reconnect the disparity between the evaluation performed when training a neural network and the evaluation of the overall communication system. The results experimentally validated the capabilities of the method, which consistently obtained the lowest EVM when deployed in a real experimental setup, using orthogonal frequency division multiplexing (OFDM) and spectrally efficient frequency division multiplexing with a varying bandwidth. S. Ullah et al. [9], in the paper on “*A Comprehensive Open-Source Simulation Framework for LiFi Communication*”, provides an open source for fully functional access points equipped with the physical layer and medium access control, a mobility model for the user device, and integration between LiFi and WiFi, with handover capabilities.

Optical camera communications (OCC) have been increasingly considered, both as a commercial product and as research, due to the availability of cameras in daily activity, through their implementation in mobile devices, industrial cameras, etc. This area is covered by the following six papers. V. Guerra et al. [10], in their paper on “*Characterization and Performance of a Thermal Camera Communication System*”, focus on a comprehensive characterization of the Peltier–Thermal camera pair in terms of bandwidth, achievable data rate using on-off-keying (OOK) modulation, and considering noise characteristics and energy efficiency. A comparison against the current state-of-the-art OCC technology is also provided, showing that TCC is a promising technology suitable for sensor networks. In the next paper on “*Optical Camera Communications for IoT–Rolling-Shutter Based MIMO Scheme with Grouped LED Array Transmitter*”, S. Teli et al. **[11]** investigate the rolling shutter-based multiple-input multiple-output OCC scheme for the Internet of Things (IoT) applications. A simplified design of multi-channel transmitter using small 8 × 8 distributed LED array, based on grouping of LEDs, is proposed and tested for flicker-free transmission. C. Jurado-Verdu et al. [12], in their paper on “*Optical Camera Communication as an Enabling Technology for Microalgae Cultivation*”, show how the OCC technology can be used as a potential application in microalgae production plants. Authors proposed a proof-of-concept prototype consisting of an artificial lighting photobioreactor, which optimizes the culture’s photosynthetic efficiency, while transmitting OOK signals to a rolling-shutter camera and analyzing node arrangements in a real facility, considering node visibility, channel capacity, and space exploitation. In the next paper on “*Wireless Sensor Networks Using Sub-Pixel Optical Camera Communications: Advances in Experimental Channel Evaluation*”, V. Matus et al. [13] propose a unique utilization of the novel topology of sub-pixel projection of multiple transmitters over the receiver, using small optical devices as a solution in OCC, i.e., re-using the camera for communication purposes as well as video-monitoring, and providing results from testing within an outdoor wireless sensor network. O. I. Younus et al. [14], in the paper on “*The Utilization of Artificial Neural Network Equalizer in Optical Camera Communications*”, outline more in-depth methods of signal processing within OCC. An artificial neural network-based equalizer is proposed and experimentally evaluated for the constant power 4-level pulse amplitude modulation, demonstrating improvement in the OCC system performance. A. Mederos-Barrera et al. [15], in the paper on “*Design and Experimental Characterization of a Discovery and Tracking System for Optical Camera Communications*”, focus on the real-time detection of the source within the image. The proposed receiver architecture combines discovery and tracking algorithms that analyze spatial features of a custom RGB LED transmitter matrix, highlighted in the scene by varying the cameras’ exposure time.

Another very promising research area within VLC is visible light positioning (VLP), which allows one to reach precision positioning in terms of centimeters, which is a tremendous advance, especially in an indoor environment, as compared to the traditional RF-based positioning systems. S. Bastiaens et al. [16], in the paper on “*A Comprehensive Study on Light Signals of Opportunity for Subdecimetre Unmodulated Visible Light Positioning*”, discuss signal processing using the LED’s characteristic frequency as a discriminative feature in photodiode-based received signal strength (RSS) unmodulated VLP. W. Raes et al. [17], in the paper on “*Experimental Evaluation of Machine Learning Methods for Robust Received Signal Strength-Based Visible Light Positioning*”, provide an experimental evaluation of machine-learning methods for robust RSS-based VLP. The methods use relative RSS-based input features to provide a more reliable localization system, when unknown changes in the received signal strength occur due to PD aperture obfuscation or LED dimming. In the next paper on “*An Indoor Visible Light Positioning System Using Tilted LEDs with High Accuracy*”, by N. Chaudhary et al. **[18]**, a specific approach with the transmitters oriented towards the center of the receiving plane (i.e., the pointing center F) is proposed, when the received power level is maximized due to the line-of-sight components on the focal point. The proposed scheme offers a significant accuracy improvement of up to ~66%, as compared to a typical non-tilted transmitter VLP, at a dedicated location within a room, using a low complex linear least square algorithm with polynomial regression. In the last paper on “*Impact of Transmitter Positioning and Orientation Uncertainty on RSS-Based Visible Light Positioning Accuracy*”, N. Chaudhary et al. [19] present simulation-based results on the impact of the transmitter’s position and orientation uncertainty, on the accuracy of the VLP system based on RSS. Authors focus on several constraining factors for RSS-based algorithms, particularly multipath channel characteristics and set-up uncertainties.

The last three papers are devoted to applications of VLC within underwater optical communications (UWOC) and vehicular communications. H. Jiang et al. [20], in the paper on “*LDPC-Coded CAP with Spatial Diversity for underwater visible-light communication (UVLC) Systems over Generalized-Gamma Fading Channel*”, propose a low-density parity-check (LDPC)-coded CAP modulation with spatial diversity, to mitigate turbulence-induced fading in a UVLC channel. Authors derive an approximated bit-error rate (BER) for the CAP modulation scheme, with a spatial diversity and equal-gain combination. They also simulate the performance of the CAP system with the diversity and LDPC for various turbulence conditions. T. Essalih et al. **[21]**, in their paper on “*Optical OFDM for SiPM-Based Underwater Optical Wireless Communication Links*”, investigate the use of high spectral efficiency modulation schemes, based on orthogonal-OFDM, namely DC-biased, asymmetrically-clipped, and layered asymmetrically clipped O-OFDM (DCO-, ACO-, and LACO-OFDM), and discuss the limitations in terms of the silicon photo-multiplier-based Rx saturation and the limited dynamic range of the transmitter, which determine the range of operation of the UWOC link, for a given target BER. In the last paper, “*Performance of Vehicular Visible Light Communications under the Effects of Atmospheric Turbulence with Aperture Averaging*”, E. Eso et al. **[22]** investigate the performance of a vehicular VLC link with a non-collimated and incoherent light source (a light-emitting diode) as the transmitter, and two different optical receiver types—a camera and photodiode—under the atmospheric turbulence conditions, and by considering the aperture averaging feature.
